# Isolation and screening of native ureolytic bacteria that induce calcium carbonate precipitation for prospective use in construction materials

**DOI:** 10.1007/s13205-026-04771-6

**Published:** 2026-04-28

**Authors:** D. P. Tamayo-Figueroa, J. Lizarazo-Marriaga, P. F. B. Brandão

**Affiliations:** 1 Instituto de Biotecnología (IBUN), Facultad de Ciencias, Universidad Nacional de Colombia–sede Bogotá, Cr. 30 #45-03, Bogotá, Colombia; 2 Grupo de Estudios para la Remediación y Mitigación de Impactos Negativos al Ambiente (G.E.R.M.I.N.A.), Laboratorio de Microbiología Ambiental y Aplicada, Departamento de Química, Facultad de Ciencias, Universidad Nacional de Colombia–sede Bogotá, Cr. 30 #45-03, Bogotá, Colombia; 3 Grupo de Investigación en Estructuras y Materiales (GIES), Departamento de Ingeniería Civil y Agrícola, Facultad de Ingenieria, Universidad Nacional de Colombia–sede Bogotá, Cr. 30 #45-03, Bogotá, Colombia

**Keywords:** *Glutamicibacter*, Urease, MICP, Cement-based materials, Calcite.

## Abstract

Microbiological induced calcite precipitation (MICP) was evaluated through the isolation, characterization, and selection of ureolytic bacteria with potential application in cement-based materials. A total of 50 bacterial strains, including newly isolated and previously reported isolates, were screened in a two-phase selection process based on urease activity and calcium carbonate precipitation efficiency. Ureolytic bacteria were isolated from cement-based materials collected from five locations in Colombia, and identified by 16S rRNA gene sequencing, revealing representatives of the genera *Arthrobacter*, *Bacillus*, *Staphylococcus*, *Glutamicibacter*, *Rhodococcus*, *Psychrobacillus*, and *Chungangia*. Notably, *Glutamicibacter* and *Chungangia* are reported for the first time as calcite-precipitating bacteria in the context of cement-based materials. Eleven strains precipitated more than 99.7% of the supplied calcium (≈ 25 mM) within 24 h in urea–Ca(NO₃)₂ medium. X-ray diffraction analysis confirmed calcite as the predominant calcium carbonate polymorph in all preselected isolates. Comparative evaluation of ammonium production, pH increase, and biomass formation demonstrated strong strain-dependent metabolic responses. A quantitative ranking approach based on normalized urease activity and precipitation efficiency enabled the selection of four candidate strains representing distinct genera. *Glutamicibacter arilaitensis* M3C3 and *Psychrobacillus psychrodurans* S17 exhibited the highest urease activity and ammonium production, whereas *Arthrobacter crystallopoietes* M4C20 and *Rhodococcus qingshengii* S1 achieved complete calcium precipitation with markedly lower urease activity. These results highlight the metabolic diversity of ureolytic bacteria associated with cement-based materials and identify novel native strains with contrasting biomineralization strategies. The selected isolates represent promising candidates for further evaluation in biocementation and self-healing applications, particularly where rapid precipitation and reduced ammonium release are desirable.

**Supplementary Information:**

The online version contains supplementary material available at 10.1007/s13205-026-04771-6.

## Introduction

Microbiologically Induced Carbonate Precipitation (MICP) is a process that occurs naturally in different environments, where mineral biogenic precipitation is carried out by different types of organisms and microorganisms, such as plants, bacteria, fungi and protists (Seifan and Berenjian 2019). Through this process, the metabolic activity of microorganisms drives the precipitation of minerals such as calcium carbonate. One of the most studied metabolic activities is the precipitation of calcium carbonate through ureolytic bacteria, since significant amounts of the mineral are deposited during this process in short periods of time (Gorospe et al. 2013; Kim et al. 2015; Krajewska 2018; Gomez et al. 2019; Almajed et al. 2021; Arpajirakul et al. 2021). This process is crucial in various biotechnological applications, such as biocementation and bioremediation, where microbial activity induces the precipitation of calcium carbonate, thereby contributing to soil stabilization or the repair of concrete structures (Al-Thawadi 2011; Hammes and Verstraete 2002; Kaur et al. 2012).

Research on the use of MICP microorganisms in the construction industry has increased significantly in recent decades (Hu et al. 2025), where the incorporation of bacteria into mixing or repairing processes aims to act as a biocatalyst to induce the precipitation of calcium carbonate in pores and cracks; as a result, porosity is reduced, while density and mechanical properties are increased. This improves the structure’s durability and load-bearing capacity, resulting in reduced water absorption and vulnerability to weather conditions, thus extending its service life, and reducing the need for frequent infrastructure repairs (Ruan et al. 2019; Kaur et al. 2020; Luhar et al. 2022; Garg et al. 2022; Nasser et al. 2022).

The incorporation of *Sporosarcina pasteurii* (halophilic, alkalophilic, endospore-forming bacteria) has been widely reported and studied in biomineralization processes applied to the construction industry due to its high urease activity, non-pathogenicity, and tolerance to high concentrations of ammonium (Gorospe et al. 2013; Lauchnor et al. 2015; Omoregie et al. 2016, 2019; Ma et al. 2020). One approach to developing the MICP process has been the isolation and identification of ureolytic bacterial strains from natural or built environments (Montaño-Salazar et al. 2018), including limestone caves (Omoregie et al. 2016), concrete samples (Vahabi et al. 2015), local soils (Diez-Marulanda and Brandão 2023), and activated sludge (Yang et al. 2020), among others. Some bacteria recovered from natural environments have shown high urease activity and potential use in biocementation processes, such as Rohmah et al. (2021) *Bacillus* sp. showing greater growth activity than *S. pasteurii* and *S. ureae* during MICP (Zhu et al. 2024).

The search, selection and characterization of bacteria carried out using MICP is one of the key aspects to improve the use and possible application of this biotechnology in the construction materials industry (Farajnia et al. 2022). The effectiveness of these microorganisms in materials under environmental conditions depends on bacterial metabolism, the adaptability of microorganisms to these conditions (native microorganisms), and abiotic factors in the environment. Specifically, for cement-based material applications, the isolated bacteria must exhibit urease activity under conditions such as high alkalinity (pH > 9), low humidity, and nutrient deficiency without affecting their MICP capacity at higher ammonium concentrations.

One of the biggest challenges to fully take advantage of this biotechnology is discovering increasingly efficient bacteria; therefore, it is essential to identify and isolate microorganisms with better MICP capabilities, suitable for construction applications. Microbial diversity is vast, and unexplored opportunities exist in terms of organisms with ureolytic capacity. The search for new strains expands the possibilities and likelihood of finding organisms with accelerated precipitation rates, strong colonization capacity, and resistance to adverse conditions.

The variation in precipitation activity among different strains represents a promising opportunity for the isolation and identification of previously unreported MICP microorganisms. Identifying novel microbial strains can provide insights into the molecular and biochemical mechanisms underlying MICP, contributing to a greater understanding of microorganism-mineral interaction in natural and artificial environments. More effective and efficient microorganisms could lead to the optimization of this biotechnological process for targeted applications, such as the repair and enhancement of different construction materials. It is important to note that the present study is framed within a passive MICP application, where microbial activity occurs in an externally applied medium prior to being long-exposed to the highly alkaline cement matrix, and long-term bacterial viability at concrete pH is not required. Thus, this research aimed to isolate, characterize, and select urease-producing bacteria capable of precipitating calcium carbonate in less than 24 h.

## Methods

The process for characterizing and selecting ureolytic bacteria was conducted in two phases and included 50 strains: 32 strains newly isolated in this study, 17 previously reported strains (Montaño-Salazar et al. 2018), and the control strain *Arthrobacter crystallopoietes* KNUC403 (Park et al. 2010).

Phase I involved the characterization of the bacteria from the MICP collection, including crystal formation analysis and molecular identification. Phase II consisted of a preselection using Urea-Ca(NO_3_)_2_ broth, followed by selection in TSB-Urea broth without calcium.

The main criteria for selecting promising isolates were ammonium production (as an indicator of urease activity), calcium ion concentration in the medium, and bacterial growth. The methodologies for each phase are described below.

## Chemical reagents

All reagents were of analytical grade. Stock solutions of calcium (1.27 M, Ca(NO₃)₂·4 H₂O, PanReac AppliChem, Germany) and urea (500 g/L, ChemCruz, USA) were sterilized by filtration (0.22 μm, Sartorius Biolab Products, Germany). Culture media were sterilized by autoclaving (121 °C, 15 psi, 20 min). Filter-sterilized calcium and urea were added under aseptic conditions.

## Phase I: isolation and initial characterization

### Sampling and isolation

Bacteria were isolated from limestone blocks, cement, and concrete samples collected from different Colombian structures (Supplementary material Table S1). Sampling sites were selected based on the type of construction material, exposure to various environmental conditions, and sample accessibility. Approximately 10 g of each sample were processed in 90 ml of sterile saline solution (0.85% NaCl), incubated for 8 days at 10 °C with agitation (150 rpm), and serially diluted (10⁻¹–10⁻⁷). The dilutions were plated onto urea–CaCl₂ agar (pH 6.8) and incubated at 30 °C. Colonies showing visible crystals under stereoscopic inspection were purified, transferred to Tryptic Soy Agar (TSA) supplemented with urea (20 g/L), and stored in 20% glycerol at − 80 °C. The strain *A. crystallopoietes* KNUC403 (Park et al. 2010) was used as a control.

### Qualitative urease activity

All isolates (32 new + 17 previously reported + KNUC403) were tested in TSB (Tryptic Soy Broth, Difco) liquid medium supplemented with urea (20 g/L) and phenol red (0.012 g/L) (MacFaddin 2003). Color change from yellow to pink indicated urease activity due to ammonium release (Supplementary material Figure S1). Each test was performed in triplicate and monitored at 24, 48, and 72 h.

### Crystal precipitation in different calcium sources

To evaluate metabolic versatility, isolates were grown on media containing commercially available nutrient broth (3 g/L), NH_4_Cl (10 g/L), urea (20 g/L), NaHCO_3_ (2.12 g/L), Agar (15 g/L), and as a calcium source (at a final 25 mM concentration): CaCl₂·2 H₂O, Ca(NO₃)₂·4 H₂O, or calcium citrate. Bromothymol blue was added to citrate-containing medium at a final concentration of 0.008% (w/v) to confirm citrate utilization and pH shifts. Crystal formation was visually confirmed using a stereoscope.

### Molecular identification

Genomic DNA was extracted using the Wizard Genomic DNA Purification Kit (Promega, USA). The 16S rRNA gene was amplified using primers 27F and 1492R (Lane 1991), and single-strand conformation polymorphism (SSCP) analysis of the V4–V5 region was performed for dereplication using primers Com1 (Schwieger and Tebbe 1998) and 909R (Kato et al. 1997). At least one isolate per SSCP profile was selected for complete 16S rRNA sequencing (Macrogen, Korea). Sequences were curated with BioEdit, deposited in GenBank (Table 1), and compared with reference strains using BLASTn. Phylogenetic trees were constructed in BEAST v1.10.4 under a Bayesian framework with the GTR + G+I substitution model.

### Culture preparation

Fresh (< 24 h) colonies were grown on TSA–urea agar and transferred to TSB–urea liquid medium for overnight incubation at 30 °C. Biomass was harvested by centrifugation (8500 rpm, 15 min), washed twice in 0.85% NaCl, and adjusted to an optical density of 0.6 at 600 nm (OD_600_). Inocula were prepared at 1% v/v for all assays, which were performed in triplicate.

### Urease activity quantification

The urease activity of each isolate was determined as the amount of ammonium produced by bacterial activity, measured over a certain growth period (24 h). Ammonium concentration was measured after 24 h incubation in TSB–urea (30 g/L). A modified phenol-hypochlorite method based on the Berthelot reaction was applied (Yu et al. 2021). Due to matrix interference from TSB, culture supernatants were appropriately diluted before reaction. Reactions were incubated at 37 °C for 30 min and read at an optical density of 670 nm. Ammonium concentrations were determined by calibration curve (0.25–2 mg/L).

### Calcium quantification

Supernatants from parallel assays were analyzed with an AAnalyst300 atomic absorption spectrophotometer (Perkin Elmer, USA), equipped with a hollow cathode lamp, using the flame method at 422.7 nm. Calcium concentration was interpolated from a standard curve (0.1–5 mg/kg).

### Cell viability

Viable cells (CFU/mL) were quantified using the microdroplet plate counting method (Miles et al. 1938) on TSA agar. Dilutions up to 10⁻⁸ were plated, and colonies were counted after 24–48 h at 30 °C.

### Crystal production and characterization

Selected strains were cultured in urea–Ca(NO₃)₂ liquid medium (20 g/L urea, 25 mM Ca(NO₃)₂) without stirring at 30 °C for 15 days. Precipitates were recovered by centrifugation (8500 rpm, 15 min), washed three times with deionized water, dried at 60 °C for ≥ 2 days, and macerated before analysis.

- **X-ray diffraction (XRD)**: Performed with CuKα radiation (λ = 1.5406 Å) at 45 kV, 40 mA, 20–80° 2θ, step size 0.026°. Phase identification was done with HighScore Plus (v3.0.5) by comparison with the diffraction patterns reported in the ICSD database (Zagorac et al. 2019).
- **Scanning electron microscopy (SEM)**: Precipitates were gold-coated and imaged using secondary electron (SE) and backscattered electron (BSE) modes to assess morphology, surface texture, and compositional contrasts.

The preselection assays in Urea-Ca(NO_3_)_2_ broth were conducted under controlled conditions to evaluate the potential of selected bacterial strains for calcium carbonate precipitation. The medium consisted of urea (20 g/L) and Ca(NO_3_)_2_ (25 mM), with an initial pH of 6.8. The OD_600_ was maintained at 0.6. Incubation was carried out at 30 °C with a stirring speed of 80 rpm. Samples were taken at 0, 12, 24, and 48 h to measure variables such as ammonium concentration, pH, colony-forming units per milliliter (CFU/mL), and calcium ion concentration, following previously described methodologies.

Selection assays were performed in TSB urea broth to evaluate the bacterial strains performance under specific conditions. The medium consisted of urea (40 g/L) and TSB, with an initial pH of 6.8. The OD_600_ was maintained at 0.6. Incubation was performed at 30 °C with a stirring speed of 80 rpm. Samples were collected at 0, 12, and 24 h to measure variables such as ammonium concentration, pH, and CFU/mL, following previously described methodologies.

## Statistical analysis

All assays were conducted in triplicate and reported as mean ± standard deviation (SD). Multivariate analysis of variance (MANOVA) was used to assess the effect of strain on response variables (ammonium, calcium, CFU/mL, pH), followed by Tukey’s HSD post hoc tests. Group separation was also assessed by PERMANOVA using distance-based metrics. Data visualization (boxplots) was performed in R (Posit Team, 2025).

## Results & discussion

The process for characterizing and selecting ureolytic bacteria among the new isolates (32 strains), previously isolated strains (17 strains) (Montaño-Salazar et al. 2018), and the control strain KNUC 403 (Park et al. 2010) was carried out in two phases, with a total of 50 strains evaluated. All isolates were able to hydrolyze urea and generate calcium carbonate precipitates in urea–CaCl₂ medium, confirming their ureolytic potential (Supplementary material Table S2). The production of ammonium ions resulted in the alkalinization of the culture medium, a key biochemical indicator of MICP activity.

**Fig. 1:**
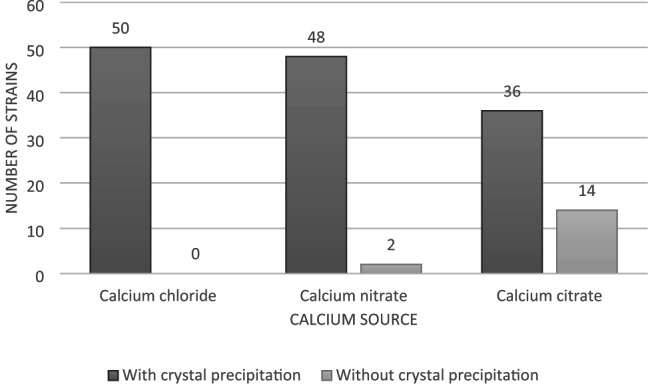
Number of strains that showed crystal precipitation with different calcium sources in media supplemented with urea. Prepared by the authors

Phase I focused on identifying the most suitable calcium source for subsequent MICP experiments. Each strain was exposed to various calcium salts to determine which supported the most efficient precipitation process. This step was essential to ensure that subsequent analyses focused on microorganisms with the greatest biotechnological potential for calcium carbonate biomineralization (Fig. 1). Although calcium chloride is commonly used in MICP studies involving cementitious materials (Bang et al. 2001 a; De Muynck et al. 2008; Achal et al. 2011; Iamchaturapatr et al. 2021), its use can accelerate corrosion of steel reinforcement due to chloride ion penetration (ACi E-701 2001; Koch 2002; Zhang et al. 2014).

**Fig. 2:**
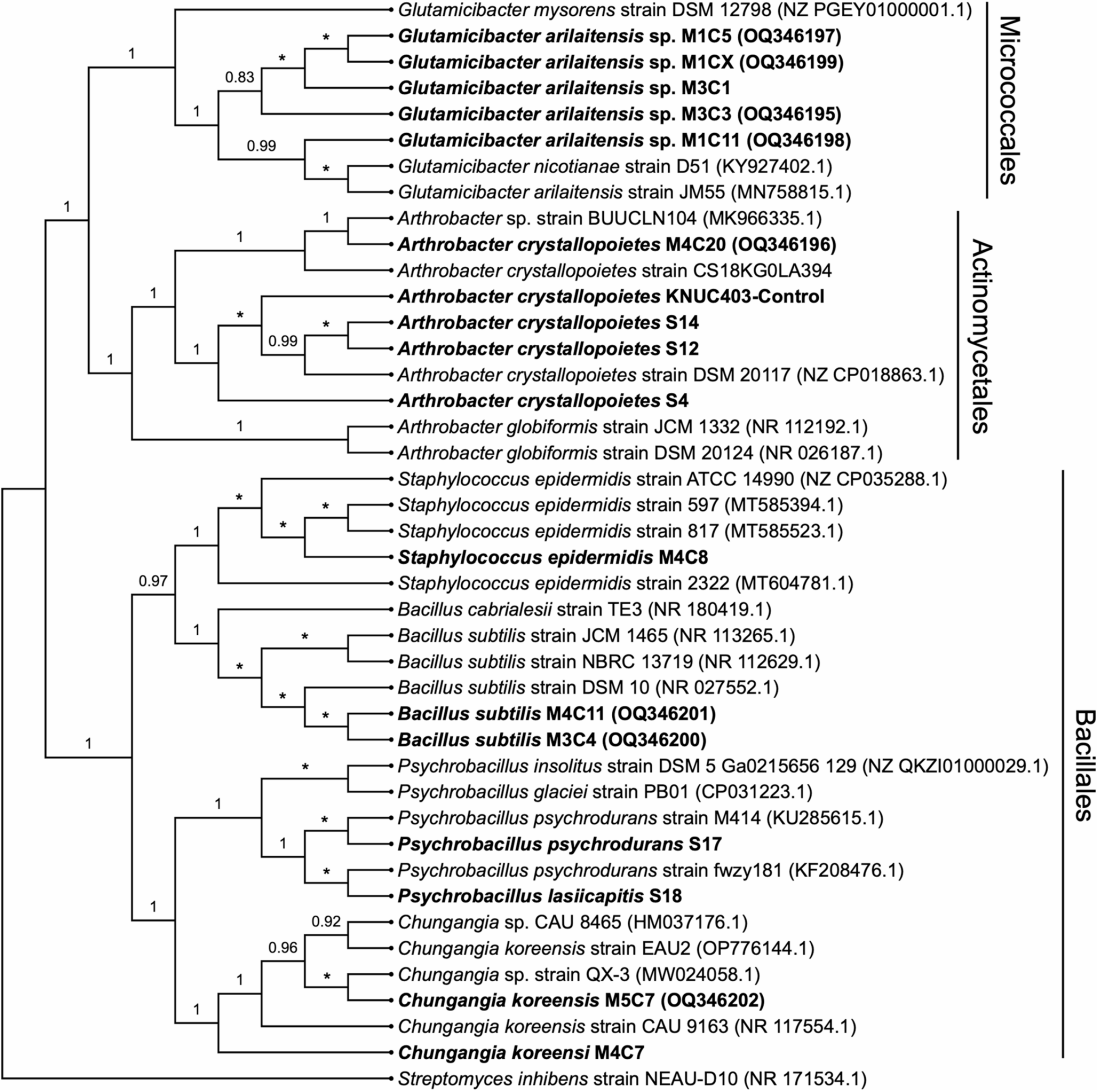
Bayesian cladogram of 16S rRNA gene sequences, representing the phylogenetic relationships of reference strains and bacterial isolates recovered from cement-based materials in Colombia. Isolates are shown in bold and correspond either to newly recovered strains (codes listed in Table 1) or to previously isolated strains (codes beginning with “S”) reported by Montaño-Salazar et al. (2018), whose equivalence and original identifiers are detailed in Table S2 (Supplementary Material). GenBank accession numbers are shown in parentheses. The 16S rRNA gene sequence of *Streptomyces inhibens* NEAU-D10 was used as an outgroup. Numbers above branches indicate posterior probability (PP) support. Asterisks denote PP support. Prepared by the authors

Most (96%) isolates produced visible precipitates in urea–Ca(NO₃)₂ medium, while only 72% did so in urea–calcium citrate medium (Fig. 2). This difference may result from the inhibitory effect of calcium ions at high concentrations, which can alter cell membrane permeability and metabolic performance (Zhang et al. 2016). This observation underscores the importance of optimizing ion balance for sustainable biomineralization performance in alkaline and calcium-rich environments such as concrete.

To reduce redundancy among isolates, SSCP analysis was used as a dereplication tool. This approach identifies unique conformational profiles of single-stranded DNA fragments (Brandão et al. 2002) allowing differentiation among strains with single nucleotide polymorphisms. SSCP analysis of the 16S rRNA gene’s V4–V5 region revealed seven distinct banding patterns (Supplementary material Figure S5), from which representative fragments were sequenced for taxonomic identification. BLAST analysis of the 16S rRNA gene sequences (*n* = 11) (Table 1) and phylogenetic reconstruction by Bayesian inference (Fig. 2) confirmed that the selected isolates belonged primarily to the Actinobacteria and Firmicutes phyla. Following the threshold suggested by Stackebrandt and Ebers (2006) and more recent reviews, a 16S rRNA gene sequence similarity of ≥ 98.7% was applied as the operational cutoff point at the species level. For instance, isolate M3C3 showed 99.3% similarity to *Glutamicibacter arilaitensis* strain JM55 (MN758815.1), while isolates M3C4, M4C20, and M5C7 were identified as *Bacillus subtilis* (100%), *Arthrobacter crystallopoietes* (100%), and *Chungangia koreensis* (99.1%), respectively (Table 1).

This analysis revealed bacteria belonging to the genera *Bacillus*, *Staphylococcus*, and *Arthrobacter*, genera previously reported as alkalophilic urease-producing bacteria, where *Bacillus* and *Arthrobacter* strains (Park et al. 2010; Zhang et al. 2018, 2020; Shaheen et al. 2021; Yang et al. 2023) have been shown to have MICP capacity and have often been used in various studies on cement-based materials.

**Table 1:** Taxonomic identification of newly recovered native bacterial strains isolated from cement-based materials, selected after SSCP dereplication and 16S rRNA gene sequence analysis, and capable of inducing calcite precipitation on urea–CaCl₂ agar

Strain	Name of the microorganisms with higher % identity^*^	Percent Identity (%)	GenBank access number of strain with higher % identity	GenBank access number of native isolate	Native isolate SSCP profile
M3C3	*Glutamicibacter arilaitensis*	99.3	NR_074608.1	OQ346195	D
M3C1	*Glutamicibacter arilaitensis*	100	AJ609625.1	PV290050	D
M3C4	*Bacillus subtilis*	100	NR_113265.1	OQ346200	C
M4C11	*Bacillus subtilis*	99.5	NR_113265.1	OQ346201	E
M5C7	*Chungangia koreensis*	99.1	NR_117554.1	OQ346202	G
M1C11	*Glutamicibacter arilaitensis*	99.9	NR_113265.1	OQ346198	D
M1CX	*Glutamicibacter arilaitensis*	99.7	NR_074608.1	OQ346199	D
M4C20	*Arthrobacter crystallopoietes*	100	NR_026189.1	OQ346196	A
M1C5	*Glutamicibacter arilaitensis*	99.6	NR_074608.1	OQ346197	D
M4C8	*Staphylococcus epidermidis*	99.9	MF428819.1	PV289772	F
M4C7	*Chungangia koreensis*	100	PP257328.1	PV248816	H

Prepared by the authors

* Name of the microorganism with the highest percentage of 16S rRNA gene identity found by BLASTn

These findings are consistent with previous MICP reports that have emphasized the role of *Bacillus*, *Staphylococcus*, and *Arthrobacter* as alkalophilic urease-producing bacteria (Bang and Ramakrishnan 2001; Bang et al. 2001; Dick et al. 2006; Vahabi et al. 2015; Schwantes-Cezario et al. 2017; Wang et al. 2017, 2019; Nain et al. 2019; Tepe et al. 2019; Reddy and Revathi 2019; Nasser et al. 2022). However, this study expands the diversity of known ureolytic taxa by including *Chungangia* and *Glutamicibacter*, marking the first report of these genera associated with MICP in cement-based environments. While *Chungangia* spp. have previously been characterized as moderately alkalophilic (optimal growth at pH ≈ 8) (Kim et al. 2012), their role in biomineralization has not been documented. Similarly, *Glutamicibacter* species are nutritionally versatile and capable of adapting to environmental stresses (Yao et al. 2015); however, no previous study has examined their involvement in urea-driven CaCO₃ precipitation. Their physiological attributes—including tolerance to salinity and alkaline conditions (Siala et al. 2015; Santos et al. 2020), suggest potential suitability for applications in cementitious environments where high pH levels and calcium concentrations prevail.

The discovery of these genera broadens the phylogenetic and metabolic framework of bacteria capable of driving ureolytic MICP, supporting the notion that taxonomic diversity may provide functional complementarity in mixed cultures, thereby improving system robustness and performance in industrial-scale applications.

Strain preselection was based on the highest urease and precipitation activity observed after 24 h of incubation. Eleven isolates (Table 2) showed the most pronounced urease activity, reflected in pH values above 9 due to ammonium accumulation, and a reduction in free calcium from 2880 mg/L to less than 7.5 mg/L. These isolates achieved calcium precipitation efficiencies exceeding 99.7%, completing the reaction approximately two-thirds faster than the reference values reported in the literature for the *Sporosarcina pasteurii* ATCC 11,859 strain (Xu et al. 2015).

**Table 2:** Factors evaluated for preselected isolates after 24 h incubation in Urea-Ca(NO_3_)_2_ medium

Strain	Name of the microorganism with higher % identity^a^	pH	Calcium (mg/L)	Precipitation (%)	Urease activity (µmol mL^− 1^ h^− 1^)
M1C5	*Glutamicibacter arilaitensis*	9.32	< 7.5	> 99.7	13.519
Positive control	*Arthrobacter cristallopoietes KNUC403*	8.23	455	84	7.960
Negative Control	*-*	8.50	1200	58	NA
M1C11	*Glutamicibacter arilaitensis*	9.72	< 7.5	> 99.7	6.314
M1CX	*Glutamicibacter arilaitensis*	9.70	< 7.5	> 99.7	13.560
M3C1	*Glutamicibacter arilaitensis*	9.81	< 7.5	> 99.7	15.670
M3C3	*Glutamicibacter arilaitensis*	9.91	< 7.5	> 99.7	31.553
M4C10	*Arthrobacter crystallopoietes*	9.21	7.5	> 99.7	15.129
M4C20	*Arthrobacter crystallopoietes*	9.48	< 7.5	> 99.7	0.993
S1	*Rhodococcus qingshengii*	9.77	< 7.5	> 99.7	1.905
S14	*Arthrobacter crystallopoietes*	9.78	< 7.5	> 99.7	5.007
S17	*Psychrobacillus psychrodurans*	9.17	< 7.5	> 99.7	20.277
S18	*Psychrobacillus lasiicapitis*	9.14	< 7.5	> 99.7	13.418

Prepared by the authors

^a^Name of the microorganism with the highest percent identity obtained by BLASTn. Negative control corresponds to culture medium without microorganism inoculation

**Fig. 3:**
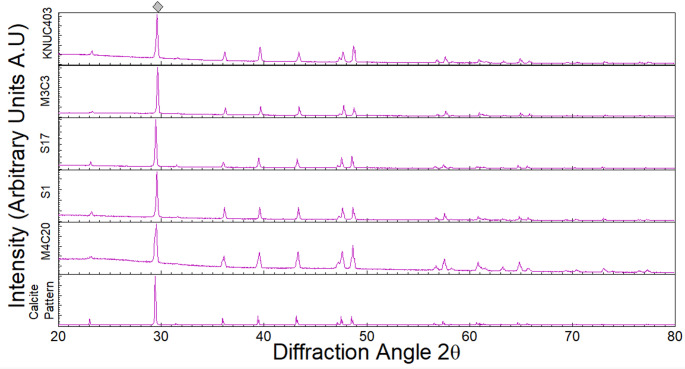
X-ray diffraction (XRD) patterns showing the characteristic diffractogram of pure calcium carbonate (reference pattern, peak at 29° below the 2-Theta scale) and the precipitated crystals obtained from four bacterial isolates after the ureolytic MICP process in urea-Ca(NO₃)₂ culture medium. Each XRD pattern corresponds to precipitates obtained from independent assays. Prepared by the authors

The mineralogy of calcium carbonate particles produced by the bacterial isolates in the urea–Ca(NO₃)₂ culture medium was analyzed using (XRD). XRD analysis was not applied to all 50 strains; instead, it was employed exclusively as a confirmatory technique to verify that the crystals formed were indeed calcium carbonate in a subset of 11 strains preselected during the initial screening stage. These strains were selected based on their superior 24-hour precipitation performance. This strategy ensured that XRD served strictly as a mineralogical confirmation tool, rather than as a selection criterion. The diffraction patterns, recorded between 20° and 80° (2θ), consistently showed the characteristic calcite peak at 29° (2θ). Representative diffraction patterns are shown in Fig. 3 (additional data is available in Supplementary material Figures S3–S4). Calcite predominance is consistent with previous reports on ureolytic MICP systems (Appanna et al. 1997; Kim and Youn 2016; Liang et al. 2022), suggesting that these isolates induce the most stable carbonate polymorph under the tested conditions.

**Fig. 4:**
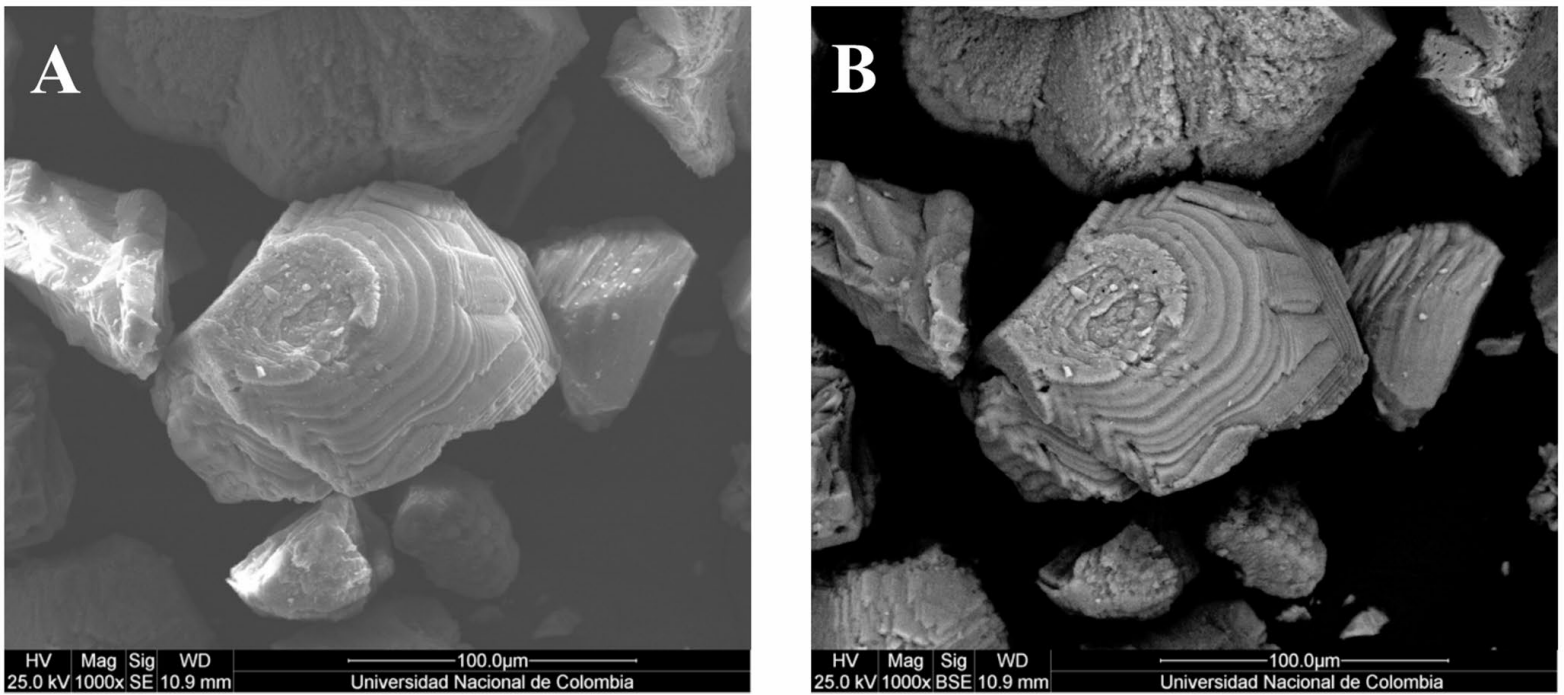
SEM images of calcite crystals produced by *Glutamicibacter arilaitensis* M3C3 in urea–Ca(NO₃)₂ medium. (A) Secondary electron (SE) and (B) backscattered electron (BSE) images at 1000× magnification (scale bar = 100 μm). Prepared by the authors

Scanning electron microscopy (SEM) images (Fig. 4A) revealed stepped and terraced crystal surfaces, features that have been previously associated with biologically influenced carbonate precipitation processes. Backscattered electron (BSE) images (Fig. 4B) showed particles with homogeneous electron density at the analyzed microscale, suggesting a uniform mineral composition within each precipitate. At the observational scale, these morphologies are consistent with microbially modulated nucleation and growth, potentially mediated by cell-surface functional groups or extracellular polymeric substances (EPS) (Rivadeneyra et al. 1998; González-Muñoz et al. 2010).

To address spatial variability and avoid overinterpreting localized features, additional SEM and BSE images acquired over larger areas (2 mm and 500 μm fields of view) are provided in Supplementary material Figure S6. These images show a relatively uniform distribution of calcite precipitates across the analyzed surface, supporting the reproducibility of the mineralization patterns observed at the mesoscopic scale.

While XRD analysis confirmed calcite as the dominant crystalline phase, it does not resolve microscale nucleation pathways. Therefore, SEM and BSE observations provide complementary evidence of crystal growth features compatible with biologically influenced precipitation. Further high-resolution in situ imaging would be required to elucidate biofilm-mediated or alternative nucleation mechanisms relevant for large-scale MICP applications (Suzana et al. 2024).

**Fig. 5:**
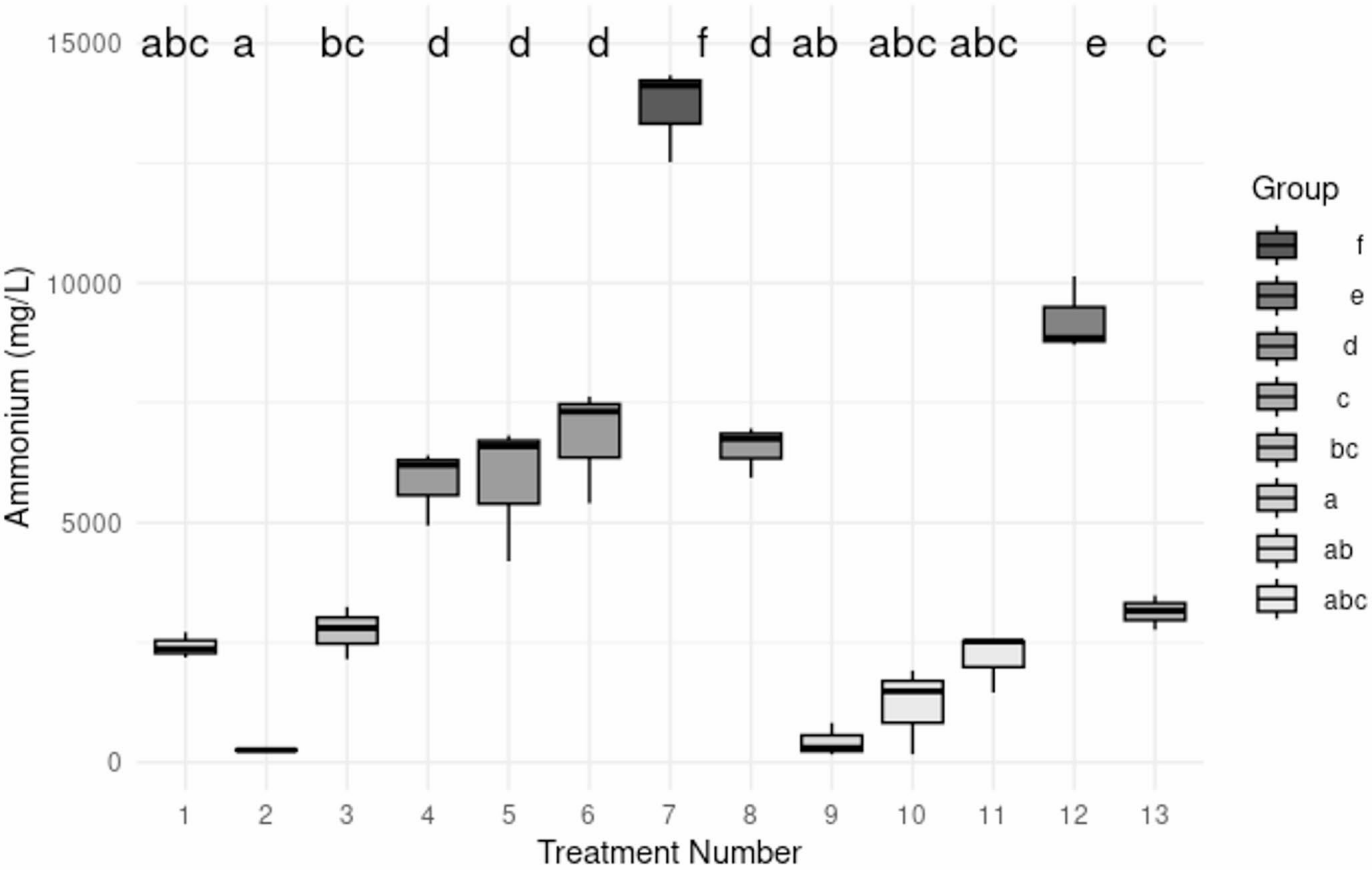
Boxplot of ammonium production (mg/L) by the 11 native isolates after 24 h of incubation in urea-Ca(NO_3_)_2_ medium. Each strain was evaluated in triplicate (*n* = 3). Positive and negative controls were also included. Numbers on the x-axis correspond to the strains as follows: (1) Positive control; (2) negative control; (3) M1C11; (4) M1C5; (5) M1CX; (6) M3C1; (7) M3C3; (8) M4C10; (9) M4C20; (10) S1; 11. S14; 12.S17; 13. S18. Different lowercase letters above the boxes indicate statistically significant differences among treatments according to Tukey’s HSD test (*p* < 0.05). Prepared by the authors

Ammonium production, pH, and optical density were monitored in urea-supplemented medium to assess urease activity in the 11 preselected isolates (Table 2). Strains M3C3 and S17 displayed the highest ammonium production over time (Fig. 5), with estimated urea hydrolysis efficiencies of 56.8% and 38.4%, respectively. MANOVA and PERMANOVA analyses confirmed specific significant differences among strains (*p* < 0.001), indicating that ureolytic activity and resulting carbonate precipitation are highly dependent on the isolate’s metabolic traits rather than on random variation. Notably, strains within the same species, such as *G. arilaitensis* M1C11 and M3C3, showed strikingly different ureolytic profiles, reinforcing the need for strain-level screening in MICP bioprospecting.

Figure 5 highlights that strains belonging to the same genus and species exhibit significantly different metabolic activities. For instance, strain *G. arilaitensis* M1C11 (position 3 in Fig. 5) produces around 3000 mg/L in 24 h, while strain *G. arilaitensis* M3C3 (position 7 in Fig. 5) produces about 14,000 mg/L during the same period. This difference is not limited to the *Glutamicibacter* genus but is also observed in isolates of the *Psychrobacillus* genus (position 12 and 13 in Fig. 5). Therefore, it can be suggested that ureolytic activity and its impact on induced carbonate precipitation are strain-specific. The identification and isolation of microorganisms with MICP capacity showed promising opportunities for applying biocementation and repairing cement-based materials. This underscores the importance of bioprospecting to expand the scope of research aimed at obtaining new isolates with demonstrable high ureolytic activity. Such endeavors can potentially yield significant benefits for diverse environmental and industrial applications.

To integrate these findings, a comparative ranking (Table 3) was generated from normalized urease and precipitation data using two composite indices: a High-performance index, combining scaled urease and CaCO₃ precipitation values to identify strong biomineralizers, and a Low-urease index, emphasizing efficient precipitation with minimal ammonium release. Both indices were derived from z-score–standardized data, with final selection ensuring taxonomic diversity across genera. The ranking identified *G. arilaitensis* M3C3, *P. psychrodurans* S17, *R. qingshengii* S1, and *A. crystallopoietes* M4C20 as the top candidates for cement-based applications (Supplementary material Figure S7).

**Table 3:** Ranking of ureolytic bacterial isolates based on normalized urease activity and calcium carbonate precipitation efficiency

Rank	Strain	Genus	Urease activity (µmol mL⁻¹ h⁻¹)	CaCO₃ precipitation (%)	High-performance index	Low-urease index
**1**	**M3C3**	***Glutamicibacter***	**31,553**	**99.7**	**0.750**	**-0.250**
**2**	**S17**	***Psychrobacillus***	**20,277**	**99.7**	**0.571**	**-0**.**713**
**3**	**S1**	***Rhodococcus***	**1905**	**99.7**	**0.280**	**0.220**
**4**	**M4C20**	***Arthrobacter***	**0.993**	**99.7**	**0.250**	**0.250**
5	M3C1	*Glutamicibacter*	15,670	99.7	0.498	0.00170
6	M4C10	*Arthrobacter*	15,129	99.7	0.490	0.0103
7	M1CX	*Glutamicibacter*	13,560	99.7	0.465	0.0351
8	M1C5	*Glutamicibacter*	13,519	99.7	0.464	0.358
9	S18	*Psychrobacillus*	13,418	99.7	0.463	0.0374
10	M1C11	*Glutamicibacter*	6314	99.7	0.350	0.150
11	S14	*Arthrobacter*	5007	99.7	0.329	0.171

The *High-performance index* and *Low-urease index* were calculated using z-score–standardized values of urease activity and CaCO₃ precipitation percentage. Strains were ranked to prioritize both biomineralization potential and taxonomic diversity. The top four strains (in bold) represent the selected candidates for further evaluation. Prepared by the authors

**Fig. 6:**
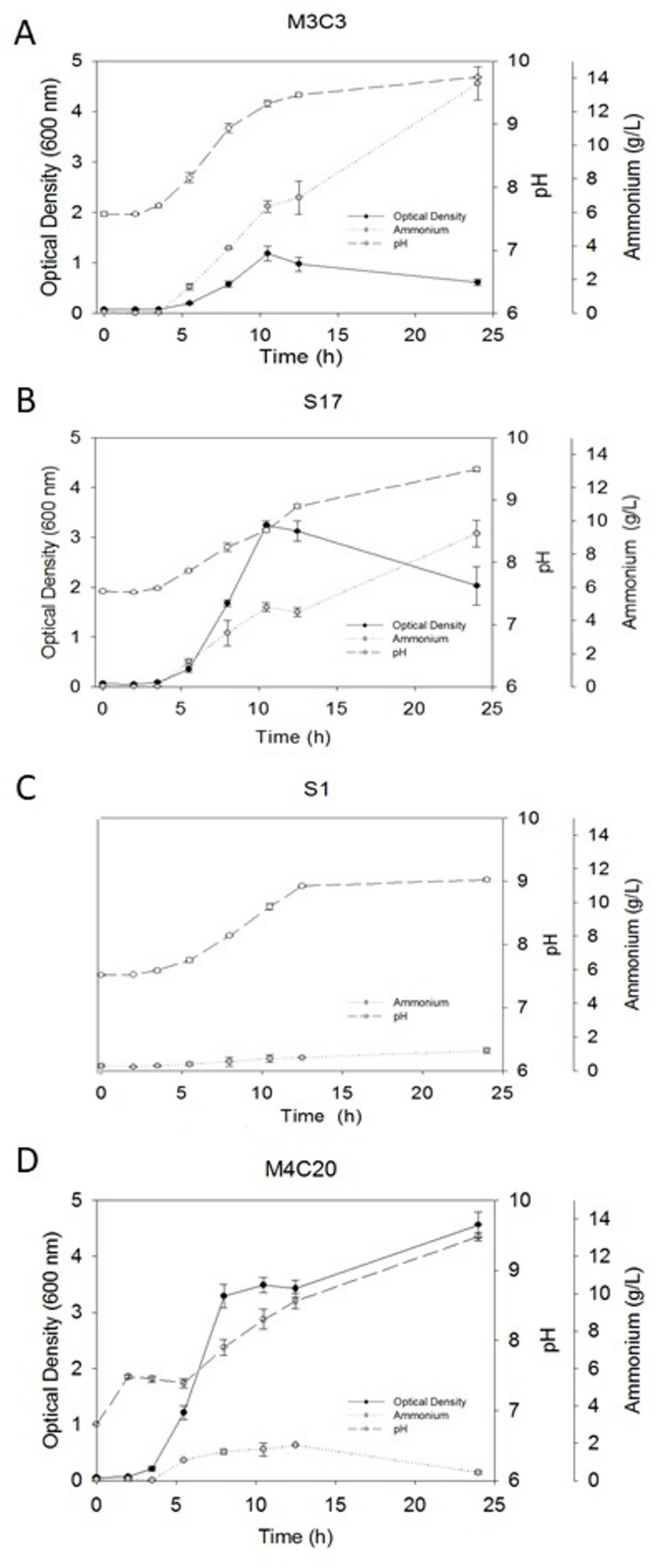
Growth curve (OD₆₀₀), pH and ammonium production (mg/L) of selected isolates over 24 h of incubation (*n* = 3). Panels represent: (A) *Glutamicibacter arilaitensis* M3C3, (B) *Psychrobacillus psychrodurans* S17, (C) *Rhodococcus qingshengii* S1 (absorbance not shown due to pellet growth), and (D) *Arthrobacter crystallopoietes* M4C20. Prepared by the authors

Strains M3C3 and S17 exhibited rapid alkalinization within 10 h of incubation (Fig. 6), suggesting that urea hydrolysis is the main precipitation driver. In contrast, *Arthrobacter* M4C20 and *Rhodococcus* S1 achieved nearly complete calcium precipitation (~ 100%) despite low urease activity, implying alternative mechanisms such as biofilm formation, EPS-mediated nucleation, or cell-surface charge modulation (Szcześ et al. 2016; Zhang et al. 2021a). This is consistent with previous evidence that non-ureolytic or low-urease strains can induce MICP via localized supersaturation zones or CO₂ hydration pathways.

Strains M4C20 and S1 proved to be organisms of particular interest for MICP applications. Although these isolates exhibited the lowest urease activity among the tested strains (Table 3), both achieved complete (≈ 100%) calcium precipitation within 24 h. This finding is remarkable, given that urease activity is generally considered a key determinant in MICP efficiency, as it drives the hydrolysis of urea into ammonium and carbonate ions, critical intermediates for calcium carbonate formation. From a practical perspective, the ability to achieve rapid and complete CaCO₃ precipitation with reduced ammonium release is highly relevant for construction-related applications, where ammonia emissions are undesirable due to toxicity, odor, and regulatory constraints in confined or urban environments (De Muynck et al. 2010).

The ranking analysis (Table 3) and composite indices further confirmed the distinctive behavior of these strains. Whereas *Arthrobacter* M4C20 and *Rhodococcus* S1 scored low in the High-performance index due to reduced urease activity, they ranked among the highest in the Low-urease index, indicating efficient CaCO₃ precipitation with minimal ammonium release. This balance is particularly advantageous for future field applications, as excessive NH₃/NH₄⁺ accumulation has been associated with secondary environmental impacts, such as nitrogen pollution and potential inhibition of microbial consortia (Seifan et al. 2016; Tittelboom et al. 2010). Low-NH₃-producing strains therefore offer a more environmentally compatible pathway for MICP-based repair strategies.

During experimentation, strain S1 displayed the formation of large aggregates or pellets that interfered with OD₆₀₀ measurements, hindering accurate biomass estimation (Fig. 6). Despite this technical limitation, its consistent and rapid CaCO₃ precipitation suggests a distinctive mineralization mode, potentially linked to its aggregation behavior. Such aggregation can enhance localized nucleation by increasing surface area and microenvironments favorable for carbonate precipitation, a phenomenon previously reported in biofilm or EPS mediated MICP system (Zhang et al. 2021b; Choi et al. 2021).

Interestingly, the ability of M4C20 and S1 to efficiently precipitate calcium despite low urease activity raises the hypothesis that these microorganisms may rely on complementary or alternative mechanisms to enhance MICP. Such mechanisms may include biofilm formation, production of extracellular polymeric substances (EPS), or modification of cell surface charge, all of which can facilitate nucleation and crystal growth. Moreover, their distinct physiological traits could make them valuable in mixed microbial consortia, where synergistic interactions between high-urease and low-urease strains optimize overall calcium precipitation while reducing ammonium accumulation, a strategy considered advantageous for scalable and sustainable construction applications (Harnpicharnchai et al. 2022).

Strain M3C3 exhibited a significant accumulation of ammonium, indicating that its primary precipitation mechanism likely involves alkalinization of the culture medium. This strain demonstrates high precipitation rates, which can be leveraged biotechnologically. Its potential for individual use as a commercial product to enhance the properties of concrete and mortar is notable, particularly for crack and fissure repair. The rapid precipitation features observed in M3C3 strain suggest a potentially competitive performance when compared to *Sporosarcina pasteurii*, based on values reported in the literature. This makes M3C3 a promising candidate for applications in construction and infrastructure maintenance, where quick and efficient calcium carbonate precipitation is essential for improving the durability and longevity of building materials.

Furthermore, the potential synergy between high-urease and low-urease strains (e.g., M3C3–S1 or S17–M4C20 combinations) could enhance MICP performance by coupling rapid alkalinization with sustained nucleation, as proposed in mixed-culture systems (Harnpicharnchai et al. 2022). Such microbial consortia can balance ammonium production, improve calcium utilization, and increase structural integration in concrete.

## Conclusions

This study highlights previously unreported cultivable microbiota in cement-based materials from Colombia, including ureolytic bacteria from genera such as *Glutamicibacter*, *Chungangia*, *Bacillus*, and *Psychrobacillus*. Out of 50 isolates, 11 precipitated 25 mM of calcium in less than 24 h, suggesting promising MICP potential compared to values reported in the literature for *Sporosarcina pasteurii.*

To our knowledge, this is the first report on *Glutamicibacter* sp. and *Chungangia* sp. isolated from cement-based environments, indicating their potential for future MICP-based applications. Further studies should evaluate the performance of these isolates in different cementitious matrices and under varying environmental conditions.

The highest urease activity and calcium carbonate formation were observed in the isolates *Glutamicibacter arilaitensis* M3C3 and *Psychrobacillus psycrodurans* S17. The ability of M3C3 to accumulate ammonium indicates that its primary MICP mechanism likely involves culture medium alkalinization, enabling efficient calcium carbonate precipitation. These features highlight its potential as a candidate for future biotechnological applications in cementitious materials.

Furthermore, noticeable intra-species variability in urease activity highlights the strain-specific nature of MICP. This supports the continued isolation and characterization of indigenous microorganisms to develop diverse microbial consortia tailored for specific applications. Notably, strains such as *Arthrobacter* M4C20 and *Rhodococcus* S1 exhibited low ammonia production.

## Supplementary Information

Below is the link to the electronic supplementary material.

Supplementary Material 1

## Data Availability

The authors confirm that the data supporting the findings of this study are available within the article and its supplementary materials.
