# Supplementary material for: Isolation and screening of native ureolytic bacteria that induce calcium carbonate precipitation for prospective use in construction materials
Source: 3 Biotech. 2026 Apr 28;16(5):176. doi: 10.1007/s13205-026-04771-6 (PMC13125588; doi:10.1007/s13205-026-04771-6)
Supplement: Supplementary file 1 — Supplementary Material 1 [file 13205_2026_4771_MOESM1_ESM.docx]

**Isolation and screening of native ureolytic bacteria that induce calcium carbonate precipitation for prospective use in construction materials**

Tamayo-Figueroa, D.P.^1-2^, Lizarazo-Marriaga J.^3^ & Brandão P.F.B.^2,*^

^1^ Doctorado Biotecnología, Instituto de Biotecnología (IBUN), Facultad de Ciencias, Universidad Nacional de Colombia – sede Bogotá, Cr. 30 #45-03, Bogotá, Colombia.

^2^ Grupo de Estudios para la Remediación y Mitigación de Impactos Negativos al Ambiente (G.E.R.M.I.N.A.), Laboratorio de Microbiología Ambiental y Aplicada, Departamento de Química, Facultad de Ciencias, Universidad Nacional de Colombia – sede Bogotá, Cr. 30 #45-03, Bogotá, Colombia.

^3^ Grupo de Investigación en Estructuras y Materiales (GIES), Departamento de Ingeniería Civíl y Agrícola, Facultad de Ingenieria, Universidad Nacional de Colombia – sede Bogotá, Cr. 30 #45-03, Bogotá, Colombia.

*Corresponding author: pfdeb@unal.edu.co

**Table S1.** Sampling locations of cement-based materials in Colombia from which samples were collected for the isolation and screening of ureolytic bacteria capable of inducing calcium carbonate precipitation (MICP). Prepared by the authors.

| Sampling site denomination | City of the sampling site | Characteristics of the sampling site | Sampling site image | Geographical coordinates |
| --- | --- | --- | --- | --- |
| M1 | Bogotá | Concrete used in drainage grates | 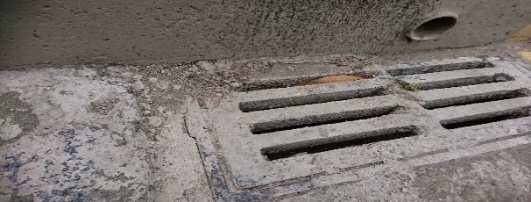 | 4°42'53.6"N 74°06'55.1"W  4.714886 -74.115300 |
| M2 | Bogotá | Lifting concrete slabs on a platform | 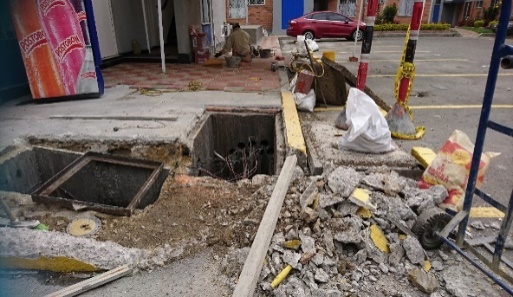 | 4°42'51.2"N 74°06'53.9"W  4.714217 -74.114984 |
| M3 | Bogotá | Cement fundation | 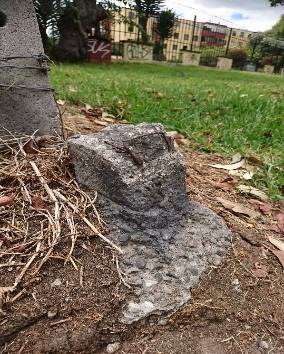 | 4°42'52.9"N 74°06'51.1"W  4.714693 -74.11418 |
| M4 | Cartagena | Fragment of limestone block with a structure similar to the walls of Cartagena obtained from Tierra Bomba Island | 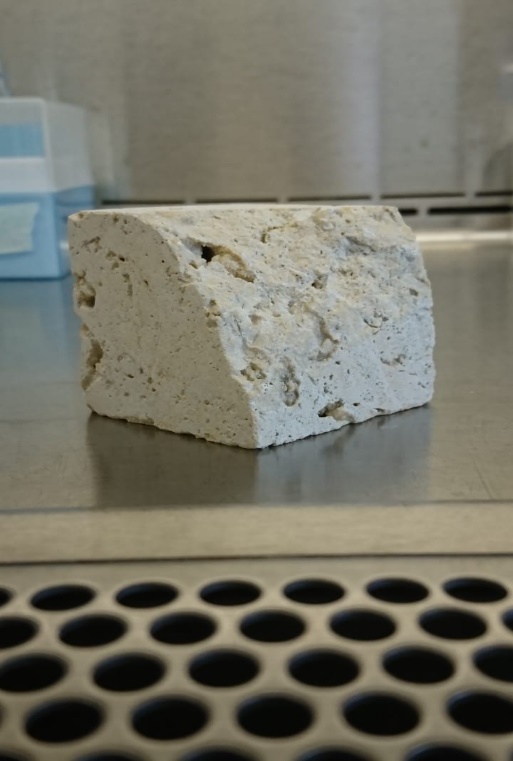 | 10°22'46.2"N 75°34'41.0"W  10.379601  -75.578059 |
| M5 | Bogotá | Concrete plate of the Transmilenio slabs | 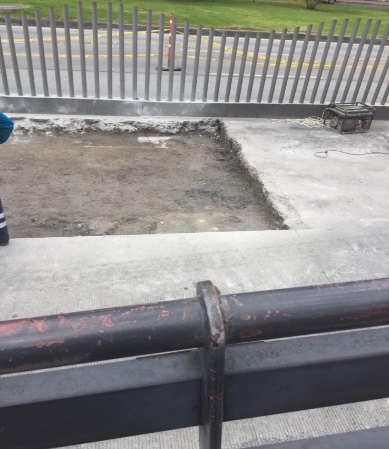 | 4°40'18.4"N 74°03'35.0"W  4.671764 -74.059719 |

**Table S2.** Physiological and molecular characteristics of the 50 isolated strains evaluated, including strains previously isolated (Montaño-Salazar et al. 2018) and those isolated during this study. Prepared by the authors.

| # | **Strain code** | **Urease cualitative assay**  **(24 h)** | **Precipitation in urea (40 g/L) CaCl_2_ (25 mM) medium** | **Precipitation in urea (40 g/L) Ca(NO_3_)_2_ (25 mM) medium** | **Use of citrate as a carbon source** | **SSCP profile**  **code** | **Code of previously isolated strains** (Montaño-Salazar et al. 2018) |
| --- | --- | --- | --- | --- | --- | --- | --- |
| 1 | **C+** | (+) | (+) | (+) | (+) | A | KNUC403 |
| 2 | **S1** | (+) | (+) | (+) | (+) | B | M101 |
| 3 | **S2** | (+) | (+) | (+) | (+) | A | M202 |
| 4 | **S3** | (+) | (+) | (+) | (+) | A | M203 |
| 5 | **S4** | (+) | (+) | (+) | (+) | A | M204 |
| 6 | **S5** | (+) | (+) | (+) | (+) | A | M205 |
| 7 | **S6** | (+) | (+) | (+) | (+) | A | M206 |
| 8 | **S7** | (+) | (+) | (-) | (+) | A | M207 |
| 9 | **S8** | (+) | (+) | (+) | (+) | A | M208 |
| 10 | **S9** | (+) | (+) | (+) | (+) | A | M209 |
| 11 | **S10** | (+) | (+) | (+) | (+) | A | M210 |
| 12 | **S11** | (+) | (+) | (+) | (+) | C | M211 |
| 13 | **S12** | (+) | (+) | (+) | (+) | A* | M412 |
| 14 | **S13** | (+) | (+) | (+) | (+/-) | C | M413 |
| 15 | **S14** | (+) | (+) | (+) | (+) | A* | M414 |
| 16 | **S16** | (+) | (+) | (+) | (+) | C | M415 |
| 17 | **S17** | (+) | (+) | (+) | (+) | C | M416 |
| 18 | **S18** | (+) | (+) | (+) | (+) | C | M417 |
| 19 | **M1C5** | (+) | (+) | (+) | (+) | D | M318 |
| 20 | **M1C5(2)** | (+) | (+) | (+) | (+/-) | D | NA |
| 21 | **M1C9** | (+) | (+) | (+) | (+) | D | NA |
| 22 | **M1C11** | (+) | (+) | (+) | (+) | D | NA |
| 23 | **M1C15** | (+) | (+) | (+) | (+) | D | NA |
| 24 | **M1Cx** | (+) | (+/-) | (+) | (+) | D | NA |
| 25 | **M3C1** | (+) | (+/-) | (+) | (+) | D | NA |
| 26 | **M3C3** | (+) | (+) | (+) | (+) | D | NA |
| 27 | **M3C4** | (+) | (+) | (+) | (+) | C | NA |
| 28 | **M3C4 (2)** | (+) | (+) | (+) | (+) | E | NA |
| 29 | **M4C4** | (+) | (+) | (+) | (+) | C | NA |
| 30 | **M4C5** | (+) | (+) | (+) | (+) | C | NA |
| 31 | **M4C5(2)** | (+) | (+) | (+) | (+) | C | NA |
| 32 | **M4C6** | (+) | (+) | (+) | (+) | E | NA |
| 33 | **M4C7** | (+) | (+) | (+) | (+) | H | NA |
| 34 | **M4C8** | (+) | (+) | (+) | (+) | F | NA |
| 35 | **M4C10** | (+) | (+) | (+) | (+) | A | NA |
| 36 | **M4C11** | (+) | (+) | (+) | (+) | E | NA |
| 37 | **M4C12** | (+) | (+) | (+) | (+) | C | NA |
| 38 | **M4C14** | (+) | (+) | (+) | (+) | C | NA |
| 39 | **M4C15** | (+) | (+) | (+) | (+) | H | NA |
| 40 | **M4C20** | (+) | (+) | (+) | (+) | A | NA |
| 41 | **M4C22** | (+) | (+) | (+) | (+) | E | NA |
| 42 | **M5C2** | (+) | (+) | (+) | (+) | C | NA |
| 43 | **M5C3** | (+) | (+) | (+) | (+) | I | NA |
| 44 | **M5C4** | (+) | (+) | (+) | (+) | C | NA |
| 45 | **M5C5** | (+) | (+) | (+) | (+) | C | NA |
| 46 | **M5C6** | (+) | (+) | (+) | (+) | C | NA |
| 47 | **M5C7** | (+) | (+) | (+) | (+) | G | NA |
| 48 | **M5C8** | (+) | (+) | (+) | (+) | C | NA |
| 49 | **M5C8(2)** | (+) | (+) | (+) | (+) | C | NA |
| 50 | **M5C9** | (+) | (+) | (+) | (+) | E | NA |

(+): positive result; (+/-): Low precipitation result. NA: Not applicable; Profile A* was designated as such because it showed a banding pattern highly similar to profile A in the SSCP gel; The rows fully shaded in gray correspond to the four strains selected after the preselection and selection tests. The cells shaded only in the "Strain code" column indicate strains that were preselected among the top 11.


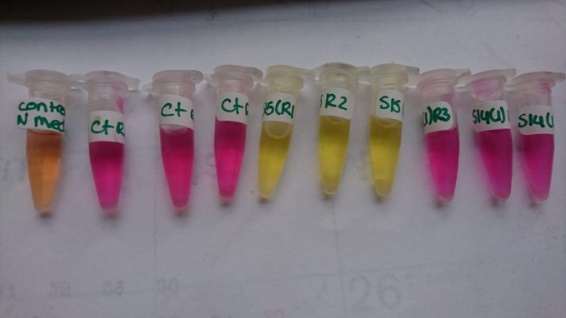


**Fig S1.** Example of biochemical assay in TSB+Urea medium to confirm urea degradation capacity by native bacteria qualitatively. From left to right: culture medium control, positive control (n=3) with *A. crystallopoietes* bacteria KNUC403, urease negative bacteria (n=3) *E. coli* ATCC 47076, urease positive bacteria (n=3) *P. psycrodurans* M414. Prepared by the authors.

| 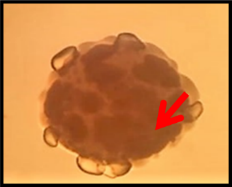  **AEsta es la nomenclatura de Sandra, pero no supe como denominar la columna** |  | 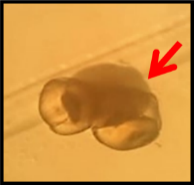  **B** |
| --- | --- | --- |

**Fig S2.** Stereomicroscopic images (1× magnification) of calcium carbonate crystals formed by *Arthrobacter crystallopoietes* KNUC403 in urea–Ca(NO₃)₂ medium at 30 °C. (A) Crystals embedded within the bacterial biomass. (B) Crystals formed at the periphery of the biomass. Red arrows indicate representative calcium carbonate crystals. Prepared by the authors


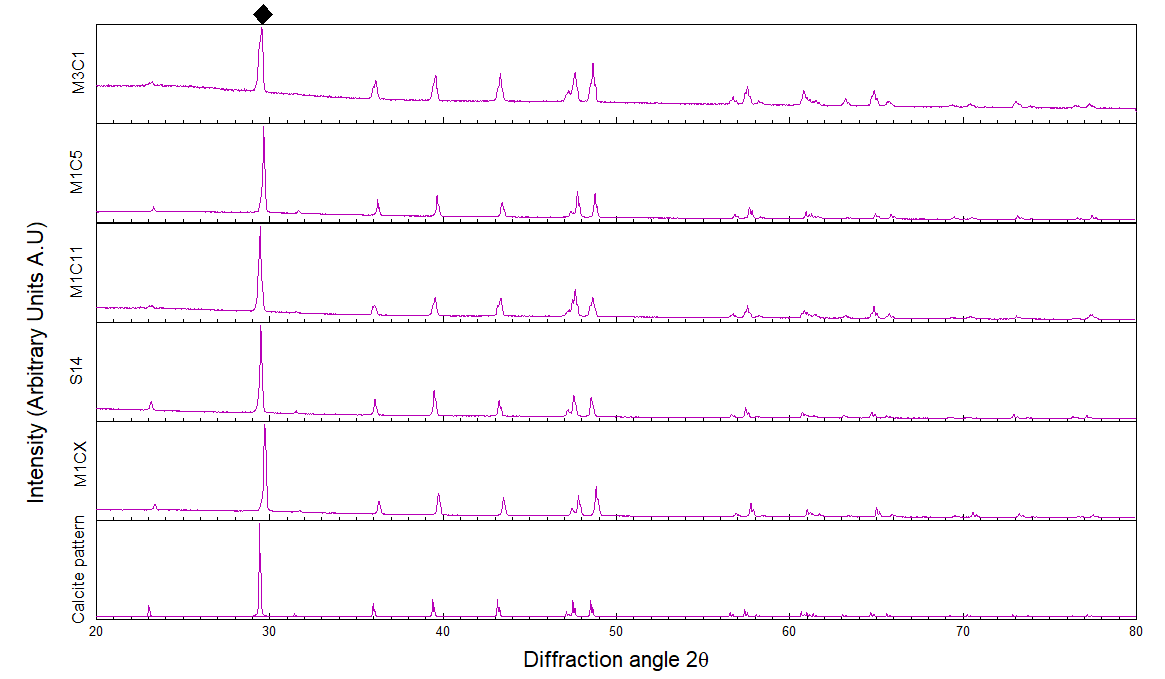


**Fig S3.** X-ray diffraction (XRD) patterns showing the characteristic diffractogram of pure calcium carbonate (reference pattern, peak at 29° below the 2-Theta scale) and the precipitated crystals obtained from five bacterial isolates (M3C1, M1C5, M1C11, S14, M1CX) after the ureolytic MICP process in urea-Ca(NO₃)₂ culture medium. Each XRD pattern corresponds to precipitates obtained from independent assays. Prepared by the authors.


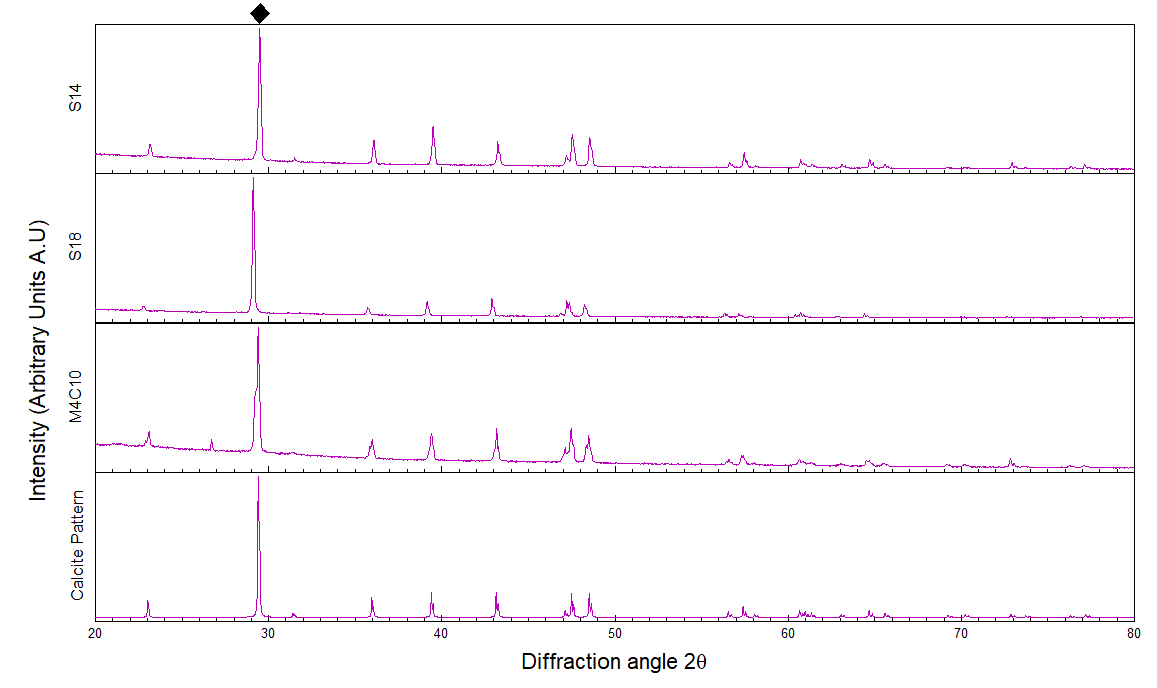


**Fig S4.** X-ray diffraction (XRD) patterns showing the characteristic diffractogram of pure calcium carbonate (reference pattern, peak at 29° below the 2-Theta scale) and the precipitated crystals obtained from different isolates (M4C10, S18, S14) after the ureolytic MICP process in urea-Ca(NO₃)₂ culture medium. Each XRD pattern corresponds to precipitates obtained from independent assays. Prepared by the authors.


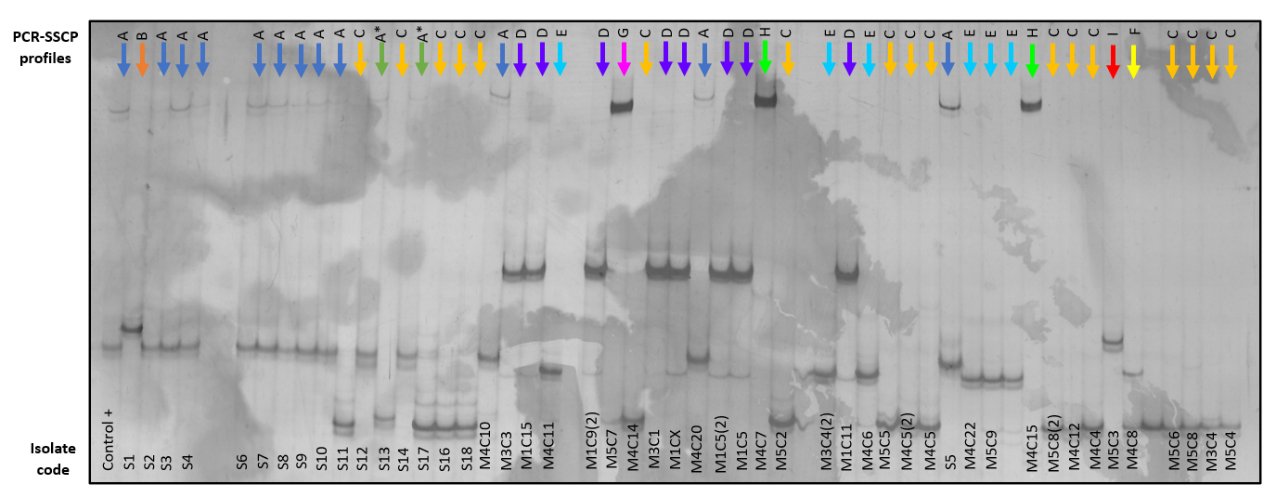


**Figure S5.** PCR-SSCP profiles of the partial 16S rRNA gene V4-V5 region of the isolates. Codes of each isolate are shown at the bottom and SSCP profile codes are at the top along with colored arrows to aid in the identification of distinct patterns. Prepared by the authors.


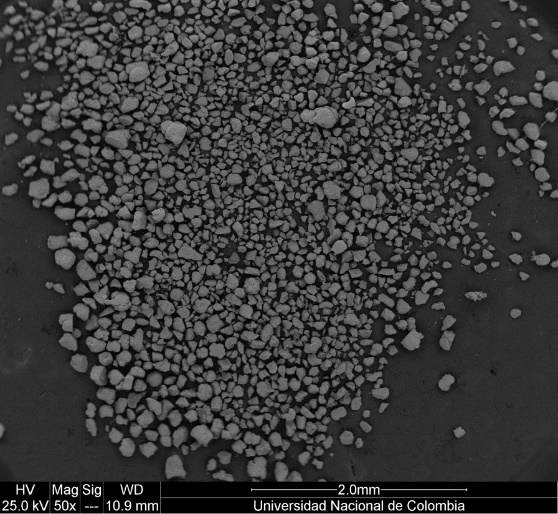

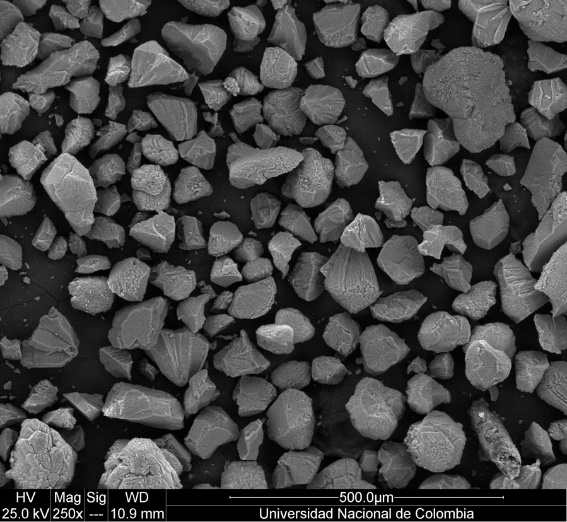


**B**

**A**

**Figure S6.** SEM images acquired using a combined secondary electron (SE) and backscattered electron (BSE) detector at two different scales 2 mm (A) and 500 µm (B), illustrating the spatial distribution and morphological uniformity of calcite precipitates formed by ureolytic bacteria during MICP. across larger areas, complementing the microscale observations shown in Figure 4. Prepared by the authors


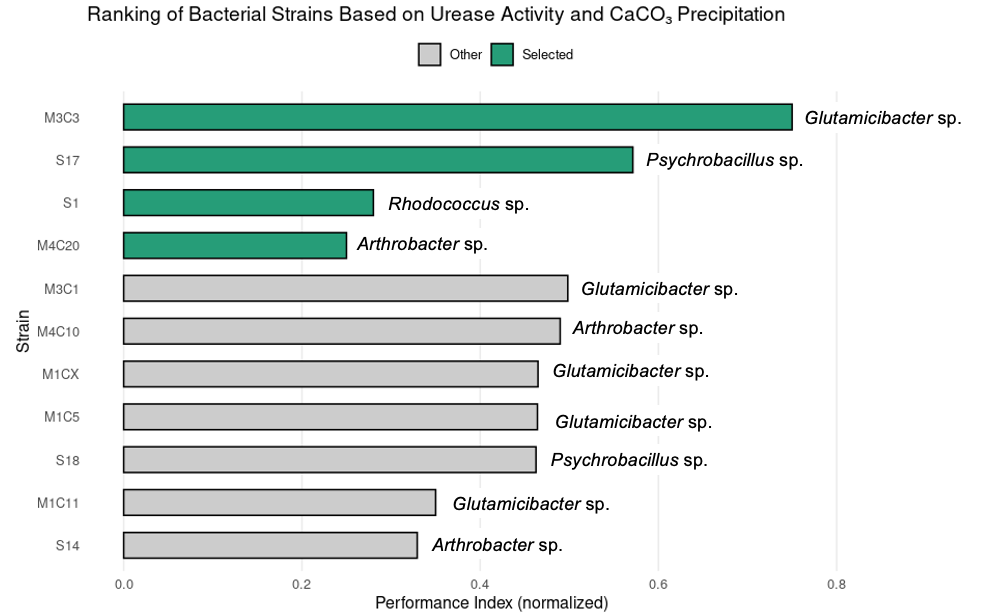


**Figure S7.** Comparative ranking of the 11 ureolytic isolates based on the two computed indices: High-performance index and Low-urease index. The visualization illustrates the relative position of each strain according to both parameters, highlighting the four selected isolates. Prepared by the authors.

**References**

Montaño-Salazar SM, Lizarazo-Marriaga J, Brandão PFB (2018) Isolation and potential biocementation of calcite precipitation inducing bacteria from Colombian buildings. Current Microbiology 75:256–265. https://doi.org/10.1007/s00284-017-1373-0
